# Game Theory Based Security in Wireless Body Area Network with Stackelberg Security Equilibrium

**DOI:** 10.1155/2015/174512

**Published:** 2015-12-02

**Authors:** M. Somasundaram, R. Sivakumar

**Affiliations:** ^1^Department of Computer Science and Engineering (CSE), R.M.K. Engineering College, Kavaraipettai, Tamil Nadu 601206, India; ^2^Department of Electronics and Communications Engineering (ECE), R.M.K. Engineering College, Kavaraipettai, Tamil Nadu 601206, India

## Abstract

Wireless Body Area Network (WBAN) is effectively used in healthcare to increase the value of the patient's life and also the value of healthcare services. The biosensor based approach in medical care system makes it difficult to respond to the patients with minimal response time. The medical care unit does not deploy the accessing of ubiquitous broadband connections full time and hence the level of security will not be high always. The security issue also arises in monitoring the user body function records. Most of the systems on the Wireless Body Area Network are not effective in facing the security deployment issues. To access the patient's information with higher security on WBAN, Game Theory with Stackelberg Security Equilibrium (GTSSE) is proposed in this paper. GTSSE mechanism takes all the players into account. The patients are monitored by placing the power position authority initially. The position authority in GTSSE is the organizer and all the other players react to the organizer decision. Based on our proposed approach, experiment has been conducted on factors such as security ratio based on patient's health information, system flexibility level, energy consumption rate, and information loss rate. Stackelberg Security considerably improves the strength of solution with higher security.

## 1. Introduction

Wireless Body Area Networks (WBANs) offer competent communication solutions to the ubiquitous healthcare systems. Smart Wearable Systems (SWS) for Health Monitoring (HM) (SWS-HM) [1] have focused on multiparameter physiological sensor systems to measure vital signs. Batch-based Group Key Management (BGKM) [2] protocol evaluates the static and dynamic conditions by placing the sensor positions at chest, head, wrist, and waist and efficiently maintains the bit error rate. Personalized e-monitoring [3] with Gaussian process helped in reducing the false alarm rate.

The proposed model accesses the patient's information with high security by applying the Game Theory based Stackelberg Security Equilibrium (GTSSE). In [4], a Signature Quality Index was designed with the objective of minimizing the error while estimating respiratory rate using WBAN. However, flexibility was compromised. The presented flexible solution in [5] is an integrated monitoring system for large scale validation using inference with kernel estimates. But this method is a basic technique in the research area of physiological monitoring. Furthermore, the proposed GTSSE over WBAN included power position authority that controls different location of the body sensors.

As in all WBANs, effective monitoring of patients is very important in a sensor network as many protocols require precise timing information. Support vector machine under ROC curve [6] and two-class classification method [7] improved the detection rate at precise time. However, to detect precise positions, significant quantities of data are required and so it is hard to design personalized setting resulting in cumbersome process. In [8], a proactive power update control algorithm was designed with the purview of providing a tradeoff between energy and network utility. In this case the technology using the Nash Equilibrium did not address security aspect which is introduced in our proposed system through Stackelberg Security Equilibrium. Remote health monitoring [9] over a long distance communication network resulted in continuous monitoring.

In this paper, we present a mechanism to access the patient's information with higher security on WBAN. This research has three main contributions:

- The first is designing a communication system which consists of WBANs that sense the information based on the game theory approach with biosensors; WBAN in our proposed system with biosensors is connected with the network to monitor multiple patients' information; local Personal Computer (PC) is connected with the network to analyze medical data stored in the server.
- The second is devising a game theory in our proposed system that provides the mathematical model for investigating multiple patients' information effectively using Stackelberg Security Equilibrium.
- Finally, the design of Birkhoff-von Neumann theorem in our proposed system diagnoses patient's health with minimal response time using permutation square matrices in the row and column to produce high security result on Wireless Body Area Network.

The rest of the paper is organized as follows.  Section 2 discusses related work.  Section 3 briefly introduces the background of the problem with the system model where steps on monitoring patient's information for WBAN are presented. The performance of our proposed mechanism is analyzed in Section 4 whereas Section 5 discusses results. Finally Section 6 concludes the paper.

## 2. Related Works

The application of WBAN is currently increasing in the healthcare applications with the improvement in the technology. In Smart Wearable Systems [1], the personal communication and healthcare assistance are provided by telematic or wearable systems. Wearable health systems improve the health monitoring action with a cost-efficient manner and reduce the direct contact of family members, friends, and care personnel. However, this system lacks the security issues. Therefore, it is proposed to use GTSSE mechanism using game theory for accessing patient's information with higher security. Ultrawideband (UWB) body sensor node mechanism is designed in [2] which offers reliable transmission of patient's information with minimum transmission power levels. But, multipath components consume more energy. Therefore, it is proposed to use proposed GTSSE mechanism that introduced Stackelberg Security Equilibrium for handling the multiple patients' health monitoring simultaneously with minimal energy consumption in Wireless Body Area Network. In [10], WBAN architecture was proposed to monitor and consult signal and health using dynamic discovery and reliable data transmission through ubiquitous healthcare profile. Multitask Gaussian process using WBAN was proposed in [11]. In this paper, the author addressed few challenges such as physiological time series, flexibility, and correlation between internal and external motion. A complete survey on artificial intelligence for public healthcare was presented in [12]. However, all the above-mentioned methods compromised information loss rate, which remains the focus of the proposed work addressed through Birkhoff-von Neumann theorem.

Game Theory Based Congestion Control Protocol in [13] addresses the congestion problem between parent nodes and child nodes in RPL-enabled networks and maximizes the throughput. However, the time consumption of packet transmission is high. In [14], the author presented a novel MAC protocol for health monitoring through WBAN. The protocol obtained different WBAN beacon frames at regular time interval for effective Wearable Health Monitoring Systems (WHMS). However, precise time synchronization was compromised. The proposed WBAN handles multiple patients' health monitoring by minimizing energy consumption using utility function. A game-based analysis on security policies developed in [15] achieves an optimal combination of security policies for content access in MSNs. However, the security, credibility, and flexibility of game based analyses are not at required level.

Recently, online anomaly detection based on WBAN has been introduced. Some utilize Haar wavelet decomposition [16] or Hampel filter for space analysis while others rely on attribute based encryption [17] mechanism to perform real time diagnosis. Both types of methods have good detection accuracy with less false alarm rate. However, error rate is not concentrated which is focused on in our proposed work by effective organizer decision using game theory approach.

In [18], distributed multiagent system was designed using WBAN for identifying human posture and detecting harmful activities. The proposed system analyzed the users' movements through physical system architecture, aiming at improving the accuracy. A complete survey on energy consumption in WBAN was presented in [19]. Fault tolerance on WBAN was introduced in [20] called visual monitoring system with the aim of taking care of the patients in a proactive manner.

All the WBAN-based techniques discussed so far are developed by providing a tradeoff between security and flexibility or considering the error rate. But, there is a need for a hybrid type of mechanism that combines the advantages of providing both a secured and low error rate system. The Stackelberg game theory provides secured data transmission as compared to other game theory approaches. So it is proposed to use proposed GTSSE mechanism that introduced Stackelberg Security Equilibrium. The work presented in this paper accesses the patient's information with higher security on WBAN, which tackles the problems of security and maintaining the error rate using Game Theory with Stackelberg Security Equilibrium (GTSSE).

## 3. Game Theory with Stackelberg Security Equilibrium Approach in WBAN

The main objective of the proposed work is to maintain the healthcare (i.e., medical information) in high security level without any threats. The maintenance is also designed with the minimal energy consumption and response time. The body sensors are wearable to identify the patient's health record and maintain it in the personal system. WBAN has exclusive architectures to adapt with the medical data applications. At each stage of the WBAN, the system is designed with the high flexibility rate with the different patient's information platforms. These sensors are used to monitor the patient's health instantly from various location points.

**Figure 1:**
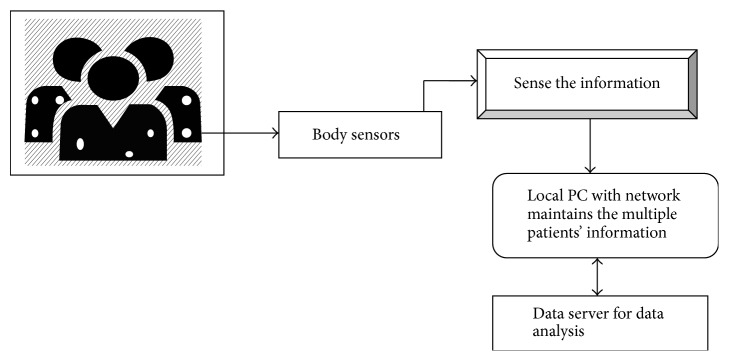
Architecture diagram of GTSSE approach.

The network architecture with the body sensor is scalable and used to effectively sense the information about the patients. The biosensors are connected with the network system to monitor multiple patients' information. Local Personal Computer (PC) is connected with the network to analyze the medical data which is stored in the server. Architecture diagram of GTSSE approach is described in Figure 1.

The three-phase work is carried out in the proposed work, where the initial phase is to sense the information based on the game theory approach with the biosensors. Second phase of the work is to establish the Stackelberg Security Equilibrium approach to maintain the security level. Third phase is to develop the Birkhoff-von Neumann theorem with “*n*” permutation matrix. The higher security level is achieved by using the interface system, where the initial phase and the third phase in GTSSE approach reduce the energy consumption rate and response time factor. The effective routing is carried out in GTSSE approach to perform the patient's information monitoring with the relay points.

**Figure 2:**
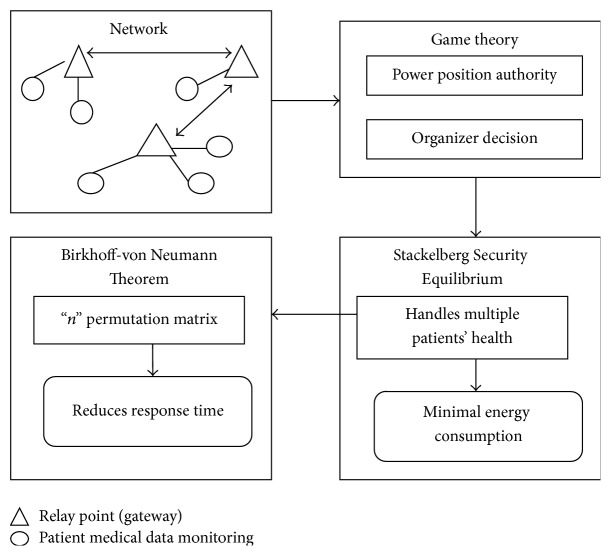
Steps on monitoring patients information in WBAN.

As demonstrated in Figure 2, game theory approach is initialized to identify the position (i.e., location) of the patients for the easier decision making process. Usually, game theory approach is the interdependent rational choice in GTSSE approach, to evaluate the patient's context behavior. The organizer is widely used in deciding the outcome of the actions in the proposed work. Game theory is used to identify the function of principles and Stackelberg Security Equilibrium is embedded in the system to handle the multiple patients' information.

Security games are developed in the wireless body sensor network based on the interaction between malicious attackers and protector. Depending on the type of the health information, the Birkhoff-von Neumann theorem helps to determine the simple and complex information statistics analysis. Birkhoff-von Neumann theorem uses the “*n*” permutation matrix which formalizes social contract and reduces the response time.

### 3.1. Game Theory Approach

Security is maintained in GTSSE approach with the relay points and using the mathematical formulations to identify the power position authority. GTSSE approach designed the system with the high security level and helps to allocate the limited resources for storing the patient's information. GTSSE approach develops the system without any jamming and attacks while using the body sensor networks. A player in the game theory approach is a basic entity of the WBAN system for making the effective decisions. A game in body sensor health monitoring provides the precise description with the strategic interaction.

GTSSE approach provides the solution to the game approach which is used in playing the best possible strategies with the effective outcome result. A policy for a game player is a complete plan of actions with all possible situations throughout the game operations to identify the patient's level of disease with the high secured framework. Game theory provides the mathematical model for investigating multiple patients' information effectively using Stackelberg Security Equilibrium, which is briefly explained in Section 3.2.

#### 3.1.1. Power Position Authority

The power position denotes the wireless network based controlling of the different location of the body sensors. The wireless sensor authority maintains the information within the particular range of space using the game theory approach. The mathematical model based position authority is formularized as

(1) $$\begin{array}{c} \text{Position}\left(\;S,J\;\right)=\overset{n}{\underset{i=1}{\sum }}\frac{\alpha_{i}S_{i}}{ps\left(\beta_{i}J_{i}\right)}. \end{array}$$

The positional points of the body sensors “*S*” are monitored and also the jamming or attacker factor “*J*” is identified on the “*i*” players. The players range from 1,2, 3,…, *n*. As *α*_*i*_ denotes the number of sensors placed within the body for the health monitoring, the position “ps” of the jammed information is identified using *β*_*i*_ in WBAN. The problem is formularized in GTSSE approach using the equality of the organizers for easy avoidance of the jammer rate.

#### 3.1.2. Construction of Organizer Decision

In GTSSE approach, game theory analyzes the behavior of different defense mechanisms type in the virtual body sensor network systems. The strategic choice is made by the organizers about the players (i.e., patients). The game model is considered with the two players, namely, the patients and organizers. The organizer identifies the players (i.e., patients) who perform the jamming performance silently in the game. It also holds the history of actions about the different players with the wearable biosensors.

The jammer creating biosensor nodes are monitored and complete information regarding the type of other nodes. The organizer in proposed work helps to reduce the consumption of energy level by easily identifying the jammer position and avoiding that in WBAN. Game theory is established to investigate the jammer position point with the biosensors using the constructed organizer decision point.

### 3.2. Stackelberg Security Equilibrium

**Figure 3:**
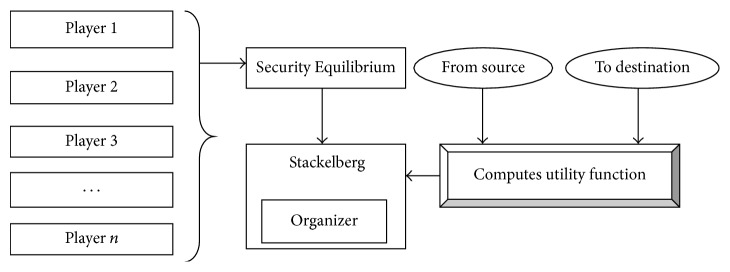
Process of Stackelberg Security Equilibrium.

In the proposed work, mathematical modeling based point of view provides the equilibrium solution over the different set of players (i.e., patients) with varying roles. The process of setting the Stackelberg Security Equilibrium is as given in Figure 3. The role of the patients is symmetric by monitoring their health information and identifies the degree of decision process. GTSSE approach has the ability to enforce the strategy Stackelberg Security Equilibrium, where the player's acts to the different location are based on the organizers. The organizers represent the leader position in WBAN, where the Stackelberg handles the multiple player (i.e., patients) information.

Stackelberg Security games in the proposed work correspond to allocating the resources for the multiple patients' body sensor information with high strategy level space. A secrecy capacity is maintained with the Stackelberg using the maximum rate of reliable information. Stackelberg Security games on multiple patient information are described as

(2) $$\begin{array}{c} SS={max}_{patients}\left(\;C_{\left(s,d\right)}-\max\left(\;C_{\left(s,d\right)}\left(\;J\;\right)\;\right)\;\right). \end{array}$$

The Stackelberg Security “SS” provides the game theory approach result based on analyzing with the multiple patients health information. The maximum patients with Stackelberg capacity from the source point to the destination *C*_(*s*, *d*)_ are identified. Then the maximal number of the jammers in that Stackelberg capacity is identified with minimal energy time “*J*” GTSSE approach. Stackelberg capacity is identified in GTSSE approach as follows:

(3) Cs,d=∑n∈NUst+Us+1t+1+Us+2t+2⋯Udt+n.

The utility function is measured on time “*t*,” where the “*N*” sensors are placed in the wireless network. *U*_*s*_ is the utility function of the source and *U*_*s*+1_ are the upcoming intermediate nodes with the patients' health information. *U*_*d*_ is the health information reaching the destination end (i.e., Personal Computer) to perform the reliable communication with the biosensors. The time gets increased as the information moves through all the intermediate nodes.

The process is repeated for all the multiple players to identify the best security factor based on the utility function. The utility function measures the resource utilization of the patient's information by the biosensors. Stackelberg uses the organizer to manage all the information. The utility function is applicable for each body sensor node to easily identify the level of jamming on the communication routing path. Stackelberg Security considerably improves the strength of solution with higher security.

### 3.3. Birkhoff-von Neumann Theorem

Each patient result is provided on the polynomial time by appealing to the Birkhoff-von Neumann theorem. Birkhoff-von Neumann theorem states that the set of “*N*” node times with “*n*” permutation matrices in GTSSE mechanism is used for diagnosing patient's health with the minimal response time. The linear size operations result in polynomial time in WBAN. The theorem is stated as follows. 

*// Birkhoff-von Neumann Algorithm*

*Begin*

*Step 1*. Initially, *n* × *n* doubly stochastic matrices forms a convex polytope with multiple player information

*Step 2*. For each row in matrix

*Step 3*. For each column in matrix set

*Step 3.1*. Non-zero real numbers are added 

*Step 3.2*. Constructed with the horizontal and vertical blocks with smaller permutation matrix

*Step 3.3*. Positive entries of the health information perform the double stochastic for the easier health diagnosis of patients

*Step 4*. Conditionally matrix is a square matrix

*Step 4.1*. Every row sums to one or all matrices entry form

*Step 4.2*. Similarly, rows points are also summed up for easier response with minimal time

*Step 5*. Wireless body sensor performs pre and post multiplication operation for the easier diagonal matrices disease identification. 

*End*

The above algorithmic code describes the Birkhoff-von Neumann theorem with “*n*” permutation matrices. The permutation square matrices sum up all the entries in the row form and also similar work is carried out on the column form to produce the high security result on the Wireless Body Area Network. The pre- and postmultiplication are also carried out on diagonal matrix for the easier computation of the time factor.

## 4. Experimental Evaluation

Game Theory with Stackelberg Security Equilibrium (GTSSE) is implemented in NS-2 simulator with the network range of 1000*∗*1000 m size. The wireless body sensors are placed to observe the patient's activities and monitor the medical records with high security level. The simulation of 25 milliseconds is taken to carry out single process. In the Random Way Point Model (RWM), each mobile node that shifts to an irregularly chosen location is considered. The RWM uses average number of mobile nodes for scheduling the nodes. The chosen location with an arbitrarily selected speed contains a predefined quantity and speed count.  Table 1 shows the simulation parameters for different performance metrics.

Dynamic Source Routing (DSR) protocol is performed in WBAN with predefined information. The GTSSE approach is compared against the existing Smart Wearable Systems (SWS) for Health Monitoring (HM) (SWS-HM) [1] and Batch-based Group Key Management (BGKM) [2] protocol. For comparison, the methods are taken from existing works but the simulation parameter values used are the same for 3 methods as given in Table 1. Also, the positions of nodes are the same for all 3 methods.

To illustrate the simulation results for GTSSE mechanism, we conducted experiments with 20 iterations and the average values are taken. The overall deviation of mobile nodes which occurred in proposed mechanism ranges from 2 to 3%.

Experiment is conducted on the factors such as security ratio based on patient's health information, energy consumption, system flexibility level based on response time, true positive rate, and information loss rate.

## 5. Analysis and Comparison

In this section, we analyze our proposed mechanism with respect to energy consumption, response time, and number of mobile nodes as well as performing the security and performance analysis. We also compare our proposed mechanism with well-known health monitoring schemes known as Smart Wearable Systems (SWS) for Health Monitoring (HM) (SWS-HM) [1] and Batch-based Group Key Management (BGKM) [2] protocol.

### 5.1. Energy Consumption

In this section, to check the efficiency of GTSSE mechanism, the metric energy consumption is evaluated and compared with the state-of-the-art works, SWS-HM [1] and BGKM [2]. Energy consumption is computed by analyzing different set of nodes (i.e., patients) and allocating resources for different nodes (i.e., multiple patients). It is measured in terms of Joules (J). The mathematical formulation for energy consumption is given as follows:

(4) $$\begin{array}{c} Energy=mobile\_nodes*\left(\;e_{players}+e_{resources}\;\right). \end{array}$$

In the above equation, mobile nodes represent the number of nodes or number of patients to be monitored during the health monitoring and “*e*_players_” represents the energy consumed for identifying different set of patients, while “*e*_resources_” is the energy consumption for allocating resources.

In order to perform a detailed experiment to understand the GTSSE mechanism which shows the results in Table 2, we apply energy consumed for different sets of patients and allocation resources to obtain the energy consumption and the comparison is made with two other existing methods, SWSHM and BGKM, respectively. Lower energy consumption results in the improvement of the framework.

**Figure 4:**
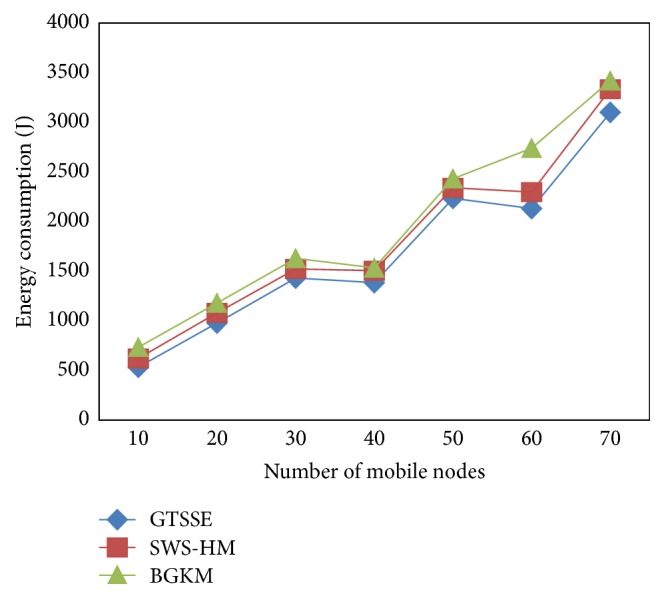
Energy consumption for three methods GTSSE, SWS-HM, and BGKM.

A comparative analysis for energy consumption with respect to different mobile nodes was performed with the existing SWS-HM and BGKM shown in Figure 4. The increasing mobile nodes in the range of 10 to 70 are considered for experimental purpose in WBAN. Initially, we consider 10 mobile nodes and the proposed GTSSE mechanism consumes 535 J energy. But, the energy consumption of existing SWS-HM and BGKM method is 623 J and 735 J, respectively. Compared to the existing state-of-the-art works, the proposed GTSSE mechanism consumes minimum energy. As illustrated in the figure, comparatively while considering more number of mobile nodes, the energy consumption rate also increases, though betterment was achieved using the proposed mechanism GTSSE.

The measurement of energy consumption is comparatively reduced using the GTSSE mechanism when compared to two other existing methods [1, 2]. This improvement in energy consumption is because of the application of Stackelberg Security Equilibrium based on the utility function of source and upcoming intermediate nodes in order to perform the reliable communication with the biosensors. Furthermore, the strategic choice is made by the organizers about the players (i.e., patients or mobile nodes) and they obtain their neighbor information in a dynamic manner reducing the energy consumption by 11.47% and 23.07% compared to SWS-HM [1] and BGKM [2], respectively.

### 5.2. Response Time

This section provides a brief description related to NS2 simulations of the proposed GTSSE mechanism as well as SWS-HM [1] and BGKM [2]. We consider DSR routing protocol of 14 mobile nodes, where a single node is implanted on the body of each patient. As our approach is application specific, we consider a specific application here, that is, Blood Pressure (BP).

We use energy model from the data sheet as shown in Table 2, whereas the response time is provided in Table 3. For this purpose, we execute our protocol 7 times and calculate response time. Response time is computed by analyzing the time taken for diagnosing multiple patients' health. The response time is formulated as given in

(5) $$\begin{array}{c} {Response}_{T}=Time\left(\;\overset{n}{\underset{s=1}{\sum }}U_{s}\;\right). \end{array}$$

In (5), the response time is the time taken to respond to multiple patients (i.e., nodes), where “*U*_*s*_” denotes time taken for diagnosing one patient's health where “*s* = 1,2,…, *n*” denotes different patients. It is measured in terms of milliseconds (ms). The lower the response time is, the more efficient the mechanism is said to be.

**Figure 5:**
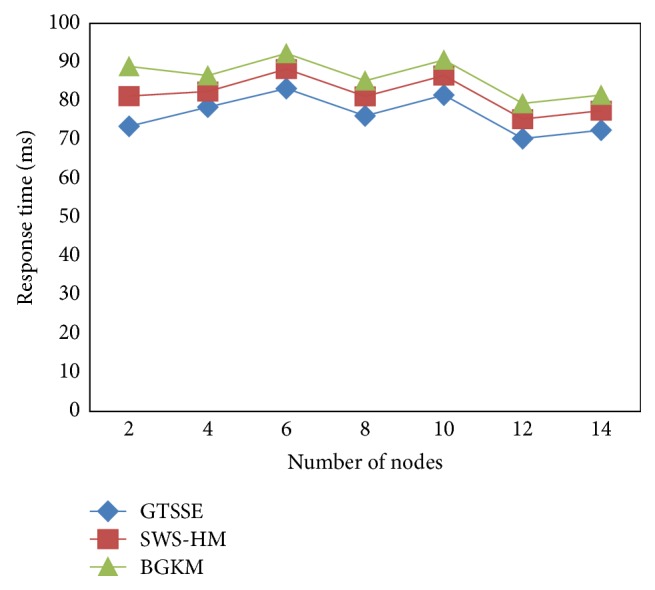
Response time for three methods, GTSSE, SWS-HM, and BGKM.

The second evaluation metric considered for evaluating the effectiveness of the mechanism GTSSE is response time with respect to different number of mobile nodes.  Figure 5 shows the elaborate comparison made with the existing two state-of-the-art works.

From Figure 5 it is clear that GTSSE performs better than SWS-HM [1] and BGKM [2]. In GTSSE mechanism, with an increase in average number of nodes, the response time also increases. As shown in Figure 5, the response time is reduced using the proposed GTSSE mechanism. With the construction of “*n*” permutation matrices, the response time is reduced using the proposed GTSSE mechanism. By constructing “*n*” permutation matrices, whenever a patient's health has to be diagnosed, Birkhoff-von Neumann in GTSSE provides polynomial time resulting in minimizing the response time. This integration of permutation square matrices in the row and column results in the improvement of response time by 7.82% compared to SWS-HM. Besides, efficient diagnosis of disease for multiple patients is performed for each mobile node through the permutation square matrices. As a result, better performance is provided and therefore the response time is reduced by 15.98% compared to BGKM.

### 5.3. Security

In order to measure the efficiency of security ratio based on patient's health information through the mechanism GTSSE, the relay points to identify the power position authority and allocation of resources are considered during the simulation in WBAN. Security in WBAN refers to the rate at which the multiple patients' health information is provided for monitoring medical information. It also refers to the ratio based on patient's health information. When the amount of security is higher, the mechanism is said to be more reliable.

The security using GTSSE mechanism is provided in an elaborate manner in Table 4 with different patients' information and simulated using NS2.

**Figure 6:**
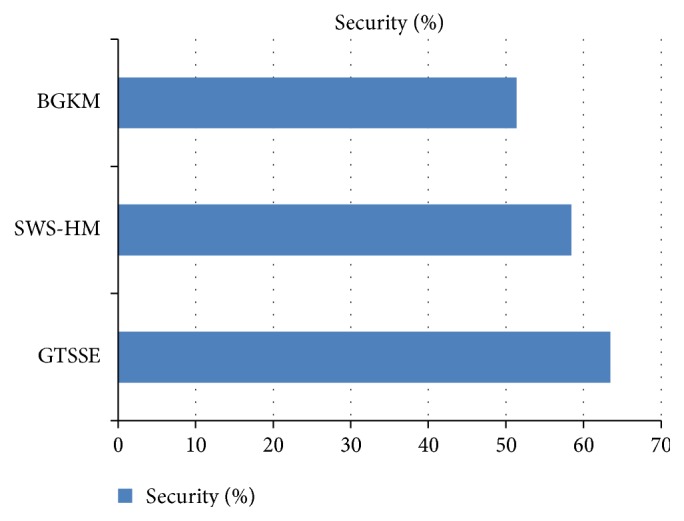
Security for three methods, GTSSE, SWS-HM, and BGKM.

Figure 6 shows the security ratio based on patient's health information with respect to 70 nodes (i.e., patients) during simulation settings at different time intervals. As depicted in the figure, with the increase in the number of nodes, the security rate is also increased. But when compared to the state-of-the-art works, the security is comparatively better than the two other methods because the proposed GTSSE mechanism uses organizer decision in WBAN and handles the information through game theory approach. So, the security is improved by 7.89% when compared to SWS-HM and 26.95% compared to BGKM, respectively.

## 6. Conclusion

WBAN contains a number of moveable, miniaturized, and independent sensor nodes to observe the body function for sporting, health, entertainment, and emergency applications. It offers long term health monitoring of patients under normal physiological states without limiting their usual behavior. In this work, an effective mechanism called Game Theory with Stackelberg Security Equilibrium (GTSSE) is presented to access the patient's information with higher security on WBAN. The goal of our game theory approach on WBAN is to sense the information with biosensors. The proposed GTSSE mechanism also identifies the power position authority which maintains the information within the particular range of space and uses the position for easy avoidance of the jammer rate. This ultimately results in the minimization of energy consumption of each mobile node and also reduces the response time while monitoring multiple patients' health information. Then, based on this measure, a Stackelberg Security Equilibrium with utility function measures not only the source but also the upcoming intermediate nodes, aiming at improving the strength of solution with higher security. Finally, we proposed a Birkhoff-von Neumann theorem for diagnosing patient's health with the minimal response time. Simulations conducted using NS2 confirm higher security with minimum energy consumption. In addition the Birkhoff-von Neumann algorithm effectively reduced the response time and information loss rate.

## Figures and Tables

**Table 1:** Simulation parameters.

Parameter	Value
Simulator	NS-2.31
Network coverage area	1000 m *∗* 1000 m
Mobility framework	Random Way Point Model (RWM)
Node movement (i.e., speed)	25 m/s
Number of nodes	10, 20, 30, 40, 50, 60, 70
Connected path link	Multidirection
Packet rate	8 packets/second

**Table 2:** Energy consumption comparison of each type of mobile nodes in WBAN using GTSSE, SWS-HM, and BGKM.

Number of mobile nodes	Energy consumption (J)		
	GTSSE	SWS-HM	BGKM
10	535	623	735
20	980	1080	1180
30	1430	1523	1631
40	1385	1505	1535
50	2235	2341	2431
60	2133	2300	2742
70	3100	3335	3421

**Table 3:** Response time comparison in WBAN using GTSSE, SWS-HM, and BGKM.

Number of nodes	Response time (ms)		
	GTSSE	SWS-HM	BGKM
2	73.5	81.23	88.9
4	78.45	82.48	86.51
6	83.21	88.23	92.26
8	76.14	81.17	85.21
10	81.55	86.55	90.53
12	70.32	75.34	79.37
14	72.45	77.48	81.51

**Table 4:** Security with respect to GTSSE, SWS-HM, and BGKM.

Methods	Security (%)
GTSSE	63.45
SWS-HM	58.45
BGKM	51.35
